# Muscle strength is associated with fracture risk obtained by fracture risk assessment tool (FRAX) in women with breast cancer

**DOI:** 10.1186/s12885-022-10203-4

**Published:** 2022-11-01

**Authors:** Rayne de Almeida Marques Bernabé, Mariana de Souza Vieira, Vanusa Felício de Souza, Luana Gomes Fontana, Ben-Hur Albergaria, José Luiz Marques-Rocha, Valdete Regina Guandalini

**Affiliations:** 1 Postgraduate Program in Nutrition and Health, Health Science Center, Federal University of Espírito Santo, Marechal Campos Avenue, 1468 – Maruípe, Vitória CEP: 29040-090 Espírito Santo, Brazil; 2 Department of Integrated Health Education, Health Science Center, Federal University of Espírito Santo, Marechal Campos Avenue, 1468 – Maruípe, Espírito Santo, Vitória CEP: 29040-090 Brazil; 3 Department of Social Medicine, Health Science Center, Federal University of Espírito Santo, Marechal Campos Avenue, 1468 - Maruípe, Espírito Santo, Vitória CEP: 29040-090 Brazil

**Keywords:** Sarcopenia, Fracture, Osteoporosis, Muscle strength, Neoplasm

## Abstract

**Background:**

Women with breast cancer are at risk for the development of sarcopenia and occurrence of fractures. The initial and periodic screening of these conditions can prevent the risks of disability, poor quality of life, and death. The present study investigated the association between sarcopenia phenotypes and fracture risk, assessed by the Fracture Risk Assessment Tool (FRAX) in women with breast cancer.

**Methods:**

Cross-sectional study. It included women aged between 40 and 80 years, diagnosed with Luminal subtype breast cancer, with time of diagnosis ≤ 12 months, who had not started endocrine therapy, did not have metastasis, had not been treated for another malignancy, and had no recurrences. Sociodemographic, habits and lifestyle, clinical, anthropometric, and body composition variables were considered. Muscle strength, skeletal muscle mass, and physical performance were investigated using handgrip strength (HGS), appendicular skeletal muscle mass index (ASMI), and Timed Up and Go test (TUGT), respectively. Fracture risk was assessed using FRAX. Multiple linear regression models were conducted to verify the association between exposure variables and sarcopenia phenotypes. A significance level of *p* < 0.05 was adopted for all tests using the SPPS 25.0 program.

**Results:**

Sixty-two women with a mean age of 58.1 ± 10.4 years were evaluated. Of these, 66.1% self-declared to be non-white, 41.9% and 71.0% did not consume alcohol or smoke, respectively, and 61.3% were insufficiently active. A total of 45.2% had clinical stage II carcinoma and 65.5% had the invasive breast carcinoma histological subtype. There was a predominance of adequacy of HGS (88.7%), ASMI (94.5%), and TUGT (96.8%), as well as low risk of hip fractures (85.5%) and major fractures (82.3%). HGS remained associated with FRAX hip fractures (*p* = 0.007) and FRAX major fractures (*p* = 0.007) in the adjusted models, while ASMI was associated with body mass (*p* < 0.001).

**Conclusions:**

Low muscle strength was the sarcopenia phenotype that remained associated with fracture risk in women with breast cancer, independently of sociodemographic factors, level of physical activity, and clinical factors. In addition to the assessment of probable sarcopenia, this measurement may point out the risk of fractures.

## Background

Women with breast cancer often experience changes in body composition related to increased fat mass and decreased fat-free mass, particularly skeletal muscle mass [1]. This condition can progress to sarcopenia, a syndrome characterized by the impairment of its phenotypes: muscle strength, muscle quantity or quality, and physical performance [2].

In individuals with cancer, sarcopenia is classified as secondary and the result of the state of systemic inflammation caused by the tumor itself and the adverse effects of anticancer treatment. Both are associated with protein catabolism and, consequently, with the reduction of muscle mass and its functions [3, 4].

Another component greatly affected in women with breast cancer is bone tissue, especially in those suffering from the subtype positive for hormone receptors (Luminal A and Luminal B) [5]. The mainstay of treatment for this subtype is antiestrogenic endocrine therapy; however, because of its systemic effects, this therapy has a strong association with bone loss and fracture risk [5].

Cancer Treatment-Induced Bone Loss (CTIBL) in women with breast cancer occurs at a faster rate than that related to age and the postmenopausal period and is related to different causes [6]. Chemotherapy, one of the most common treatments, can induce premature ovarian failure in premenopausal women and exert toxic effects on bone cells. Similarly, reversible ovarian suppressors and surgical induction of menopause (oophorectomy) are also causes of reduced estrogen levels in this population. Among postmenopausal women, aromatase inhibitors (AI), used in endocrine therapy, are responsible for suppressing peripheral estrogen production. Furthermore, patients with breast cancer often use glucocorticoids to control symptoms associated with treatment, which increases the risk of fractures [6].

Thus, early care and safe and effective interventions are necessary to preserve bone mineral density (BMD) and prevent fractures in this population [6]. Non-pharmacological care includes calcium and vitamin D supplementation and physical exercise [7]. Among pharmacological interventions, evidence suggests that the use of denosumab and zoledronic acid may be considered the most effective antiresorptive treatment options to improve BMD in breast cancer patients using AI [8].

The assessment of bone health in this population should include not only the analysis of BMD, but also the other risk factors that influence bone strength [6]. In this context, the Fracture Risk Assessment Tool (FRAX) represents an important advance in the assessment of this risk [6]. The few studies discussing the use of FRAX in women with breast cancer have concluded that FRAX scores stratify fracture risk equally well between women undergoing endocrine therapy with AI and non-users, and that all patients who start treatment with AI should have their fracture risk assessed [9, 10].

The presence of sarcopenia and increased fracture risk are conditions reported in women with breast cancer that are associated with negative outcomes in this population [10–12]. Sarcopenia is associated with shorter survival, increased chemotherapy toxicity, and faster tumor progression, while fractures are related to significant physical disabilities, high healthcare costs, increased risk of subsequent fractures, and increased mortality [13–16].

Bone and muscle tissues are linked from a biological and functional point of view [17]. In this context, sarcopenia has been investigated as a predictor of falls and fractures, since stability, balance, and the capacity of the muscle mass to absorb shock are compromised [18]. However, as sarcopenia has multiple definitions, evidence links its phenotypes to fracture risk. Previous studies carried out with elderly people observed that muscle mass is a predictor of fracture incidence, and that the decline in muscle strength and physical performance contribute independently to the increased fracture risk [19, 20].

Although women with breast cancer are at risk for sarcopenia and fractures, these conditions are little explored in this population. Early and periodic screening of both conditions can guide clinical and dietary management in order to avoid and/or reduce the chances of complications and aggravations during and after anticancer treatment. In this context, this study investigated the association between sarcopenia phenotypes and fracture risk, assessed by FRAX, in women with breast cancer.

## Methods

Observational cross-sectional study, with non-probability, convenience, and consecutive sampling, carried out in a mastology outpatient clinic of a public hospital located in Vitória, Espírito Santo, Brazil, from January 2021 to May 2022. Inclusion criteria were: to be treated at the referred outpatient clinic; to be aged between 40 and 80 years; to have a confirmed diagnosis of luminal subtype female breast cancer; to have a diagnosis time of up to 12 months; not presenting metastasis; not having started endocrine therapy; not having or having been treated for another malignant neoplasm, and not having recurrences. Women with unassisted mobility impairments, who had cognitive impairment and/or psychiatric illnesses predicted in medical records and who were under pharmacological treatment for osteopenia and osteoporosis and using calcium and vitamin D supplementation were not considered for the study.

All medical consultations performed by the mastology team were screened and the patients considered eligible were contacted by telephone – at least three attempts – and invited to participate in the research, in order to limit possible sample selection biases.

### Study variables and instruments

Data collection took place through face-to-face interviews using a structured protocol. All researchers involved were properly trained to apply the instruments and measure the parameters used.

### Outcome

The outcome variables of this study were the sarcopenia phenotypes: muscle strength, muscle quantity and physical performance. The parameters, methods, and cut-off points suggested by the Revised European Consensus on Sarcopenia [2], proposed by the European Working Group on Sarcopenia in Older People (EWGSOP), were used. Muscle strength, skeletal muscle quantity, and physical performance were evaluated using the handgrip strength test (HGS), the appendicular skeletal muscle mass index (ASMI), and the Timed Up and Go test (TUGT), respectively [2].

### Handgrip Strength (HSG)

To assess HGS, a manual dynamometer (Jamar®) with a scale from 0 to 90 kg/f and a resolution of 2 kg/f was used. The test was performed using the method recommended by the American Association of Hand Therapy (ASHT) [21]. The participant was seated, with the spine erect, the knee flexed at 90º, the shoulder positioned in adduction, the forearm supported, and the elbow flexed at 90º. The procedure was performed three times on the dominant hand, with maximum effort for about 5 s, with a 1-min interval between measurements [21]. The highest value obtained in the three measurements was considered. In cases in which the participant had undergone hand, arm or forearm surgery on the dominant side less than 60 days prior to the test, the HGS of the non-dominant hand was measured and considered. Values < 16.0 kg were indicative of reduced muscle strength in women [2].

### Appendicular skeletal muscle mass index (ASMI)

ASMI was obtained by measuring appendicular skeletal muscle mass (ASM), which consists of the sum of the skeletal muscle mass values of the upper and lower limbs, through dual energy x-ray absorptiometry (DXA). ASMI was calculated by the ratio between ASM (Kg) and height (m) squared [ASM(Kg)/height^2^(m)]. A cutoff point of ≤ 5.5 kg/m^2^ was considered to identify low skeletal muscle mass in women [2].

DXA was performed using a GE Lunar Prodigy Advance device and the GE Encoreà software, version 14.10, configured to use the reference database of the National Health and Nutrition Examination Survey (NHANES) [22]. All DXA exams were performed by the same radiology technician and reported by a single physician. The manufacturer's protocols were followed, as were the recommendations of the International Society for Clinical Densitometry (ISCD) and the Brazilian Society of Clinical Densitometry (SBDens) [23, 24].

### Timed Up and Go test (TUGT)

To perform the test, the patient was asked to get up from an armless chair unassisted, walk 3 m, turn around, return to the starting point, and sit down again [25]. This procedure was timed by the researchers and repeated three times. Physical performance was considered low when the mean time spent during the procedure was ≥ 20 s [2].

## Exposure variables

Nutritional status, fat mass compartment, and fracture risk were assessed using anthropometric variables, DXA, and FRAX, respectively.

The anthropometric data measured were: height (m), body mass (kg), calf circumference (CC) (cm), and waist circumference (WC) (cm). The body mass index (BMI) was calculated as the ratio of body mass to height squared [weight(kg)/height^2^(m)]. For adult women, the BMI was classified considering the ranges proposed by the World Health Organization (WHO) [26]. Elderly women were classified according to the cutoff points recommended by the Pan American Health Organization (PAHO) [27]. CC was measured at the largest point of the calf [28]. Values equal to or below 33.0 cm were indicative of reduced muscle mass [29]. WC was measured at the level of the umbilicus [30]. Women with values ≥ 80 cm and ≥ 88 cm were classified at high and very high risk for metabolic complications associated with obesity, respectively [26].

To assess the fat mass compartment, the values of percentage of total body fat mass (%BF) and abdominal visceral adipose tissue (VAT) obtained by DXA were considered. A %BF ≥ 32% [31] and a VAT ≥ 107.5 cm^2^ were considered high, according to the median of the population itself.

Fracture risk was identified by FRAX validated for Brazil. This tool is an algorithm calculated from age, BMI, and dichotomized risk factors, including previous fragility fracture, parental history of hip fracture, current smoking, long-term oral glucocorticoid use, rheumatoid arthritis, other causes of secondary osteoporosis, and excessive alcohol consumption. In addition, femoral neck BMD obtained by DXA was taken into account to increase the prediction of fracture risk [32]. FRAX assesses the 10-year probabilities of a hip fracture and a major fracture (hip, spine, humerus, or wrist fracture) [32]. Results are expressed as percentages and categorized into low and high risk. FRAX-Brazil is available online on the website of the Brazilian Association for Bone Assessment and Osteometabolism (ABRASSO) and can be accessed through the link: < https://abrasso.org.br/calculadora/calculadora/ > .

### Covariables

Sociodemographic data, habits and lifestyle data, menopausal status, current clinical history, and diagnostic classification of BMD were considered as covariates.

The sociodemographic variables considered were: age in years; marital status (lives with or without a partner); schooling in years of study; self-reported race/color (white, brown, black, and yellow) [33]; and occupation. Race/color was later grouped into whites and non-whites, for those who declared themselves to be black, brown or yellow.

Regarding habits and lifestyle, alcohol consumption, smoking, and physical activity level were considered. To estimate the level of physical activity, the short version of the International Physical Activity Questionnaire (IPAQ) for the Brazilian population was used [34]. This variable was classified according to the guidelines on physical activity and sedentary behavior proposed by the WHO [35]. Those who reported performing moderate physical activity for 150 to 300 min or 75 to 150 min of intense physical activity per week were categorized as “Sufficient”, while those who did not meet these criteria were deemed “Insufficient”.

Menopause status and time of menopause in years were considered. The variables time of diagnosis, clinical staging, histological subtype, and type of treatment characterize the current clinical history and were collected from the patient's medical records. The time of diagnosis was determined by the difference between the date of diagnosis in the medical record and the day of evaluation for this study. The type of treatment was classified as "No previous treatment", when the participant had not undergone treatment before the evaluation, "Neoadjuvant", for women who had only undergone neoadjuvant chemotherapy, "Surgical", for those who had only undergone surgery, and “Adjuvant”, for those who underwent adjuvant chemotherapy or radiotherapy. The histological subtype variable was categorized into “Invasive breast carcinoma”, “Invasive ductal carcinoma”, “Ductal carcinoma in situ”, and “Special subtypes”. The special subtypes found were: micropapillary ductal carcinoma, apocrine invasive breast carcinoma, micropapillary invasive breast carcinoma, pleomorphic invasive lobular carcinoma, invasive papillary carcinoma, encapsulated papillary carcinoma, cribriform ductal carcinoma in situ, and papillary ductal carcinoma in situ.

BMD was assessed by DXA in the lumbar spine (L1-L4) and right proximal femur. BMD was classified according to the lowest T-score found into: normal BMD (T-score ≥ -1), osteopenia (T-score between -1 and -2.5), and osteoporosis (T-score ≤ -2, 5) [36].

### Data analysis

The sample was characterized through descriptive analysis expressed in means and standard deviations to describe continuous variables and percentages for categorical variables. The normality of quantitative variables was assessed using the Kolmogorov–Smirnov test. The presence of correlation between sarcopenia phenotypes and continuous variables was analyzed using Pearson's Correlation. Correlation coefficients can range from -1 to + 1 and were categorized as weak (*r* < 0.3), moderate (*r* = 0.3–0.7) or strong (*r* > 0.7) [37]. Multivariate linear regression analysis (forward method) was applied to determine the influence of exposure variables on sarcopenia phenotypes (outcome variable). All variables that showed significance in the correlation test were included and raw and adjusted values are presented. The variable BMI was not included in the regression models because it presented multicollinearity after analysis of the assumptions. Adjustment variables were entered in blocks: Model 1: age (years), race/color, physical activity level; Model 2: age (years), race/color, physical activity level, menopause time (years), diagnosis time (months), clinical staging, type of treatment. Statistical analyses were performed using the Social Package Statistical Science (SPSS) version 25.0 software. For all tests, the significance level adopted was *p* < 0.05.

## Results

**Fig. 1:**
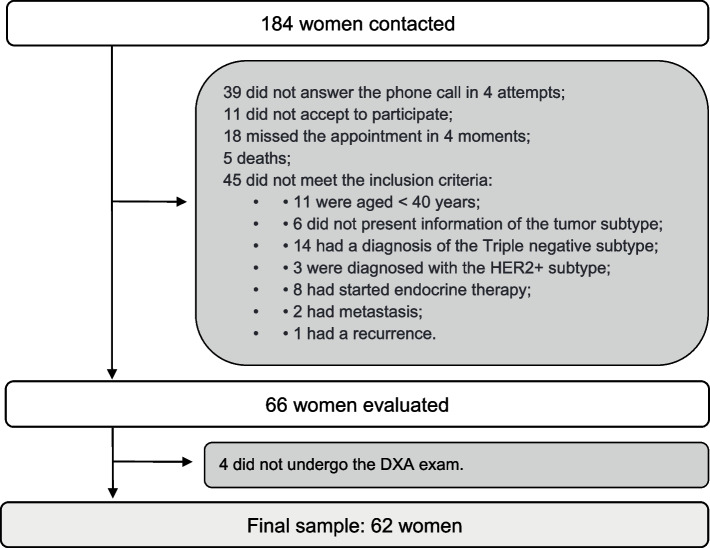
Sampling flowchart

During the study, 184 women with breast cancer, diagnosed 12 months prior to the evaluation, were seen at the outpatient clinic and contacted to participate in the research. Of those who answered the telephone contact, agreed to participate, and turned up for data collection, 45 did not meet the inclusion criteria, resulting in 66 patients evaluated in the present study. However, four did not complete data collection as they did not undergo the DXA exam, which resulted in a final sample of 62 volunteers (Fig. 1).

The women evaluated had a mean age of 58.1 ± 10.4 years, predominantly between 40–59.9 years (53.2%) (Table 1). Of these, 64.5% lived with a partner, 66.1% declared themselves to be non-white, 40.3% did not work, and 37.1% had 4 to 8 years of schooling. The vast majority never consumed alcohol (41.9%) or smoked (71.0%), in addition to being classified as insufficiently active (61.3%) (Table 1).

**Table 1:** Distribution of sociodemographic and lifestyle variables of women with breast cancer

Variables (*n* = 62)		n (%)
**Age group (years)**		
40—59.9		33 (53.2)
60—80		29 (46.8)
**Marital status**		
Lives with a partner		40 (64.5)
Does not live with a partner		22 (35.5)
**Race/color**		
White		21 (33.9)
Non-white		41 (66.1)
**Work activity**		
Does not work		25 (40.3)
Works		19 (30.7)
Retired		18 (29.0)
**Schooling (years)**		
< 4		8 (12.9)
4—8		23 (37.1)
> 8—11		21 (33.9)
> 11		10 (16.1)
**Alcohol intake**		
Never drinks		26 (41.9)
Used to drink		23 (37.1)
Drinks		13 (21.0)
**Smoking**		
Never smokes		44 (71.0)
Used to smoke		16 (25.8)
Smokes		2 (3.2)
**Physical activity level**		
Insufficient	38 (61.3)	
Sufficient	24 (38.7)	

When the clinical variables were evaluated, a predominance of clinical stage II (45.2%) and the histological subtype invasive breast carcinoma (64.5%) was observed (Table 2). There was a higher proportion of women with diagnosis time ≤ 6 months (82.3%) and who did not undergo previous treatment until the time of evaluation for this study (46.8%). The predominant menopausal status was postmenopausal (82.3%) and 52.9% of the women had a time of menopause ≤ 15 years.

**Table 2:** Distribution of clinical variables, sarcopenia phenotypes, and fracture risk in women with breast cancer

Variables (*n* = 62)	n (%)
**Clinical staging**	
0	3 (4.8)
I	20 (32.3)
II	28 (45.2)
III	11 (17.7)
**Histological subtype**	
Invasive breast carcinoma	40 (64.5)
Invasive ductal carcinoma	6 (9.7)
In situ ductal carcinoma	3 (4.8)
Special subtypes	13 (21.0)
**Diagnosis time (months)**	
< 6	51 (82.3)
6 – 12	11 (17.7)
**Type of treatment**	
No previous treatment	29 (46.8)
Neoadjuvant	10 (16.1)
Surgical	9 (14.5)
Adjuvant	14 (22.6)
**Menopausal status**	
Pre-menopause	11 (17.7)
Post-menopause	51 (82.3)
**Menopause time (years)**^**1**^	
≤ 15	27 (52.9)
> 15	24 (47.1)

^1^*n* = 51

Regarding nutritional status, there was a predominance of obese women (38.7%), with substantially increased WC (75.8%), high %BF (80.6%) and VAT (50.9%), and adequate CC (74.2%) (Table 3). Muscle strength, assessed by HGS, muscle quantity, determined by ASMI, and physical performance, measured by TUGT were mostly adequate. In addition, there was a predominance of BMD classified as normal (46.8%) and low risk of hip fracture (85.5%) and major fractures (82.3%) (Table 3).

**Table 3:** Distribution of nutritional status variables, bone mineral density, sarcopenia phenotypes, and body composition variables of women with breast cancer

Variables (*n* = 62)	n (%)
**BMI**	
Underweight	7 (11.3)
Eutrophic	14 (22.6)
Overweight	17 (27.4)
Obese	24 (38.7)
**CC**	
Adequate	46 (74.2)
Reduced	16 (25.8)
**WC**	
Adequate	5 (8.1)
Increased	10 (16.1)
Substantially increased	47 (75.8)
**%BF**^**1**^	
Adequate	5 (9.1)
High	50 (90.9)
**VAT**^**1**^	
Adequate	27 (49.1)
High	28 (50.9)
**HGS**	
Adequate	55 (88.7)
Reduced	7 (11.3)
**ASMI**^**1**^	
Adequate	52 (94.5)
Reduced	3 (5.5)
**TUGT**	
Adequate	60 (96.8)
High	2 (3.2)
**BMD**	
Normal	29 (46.8)
Osteopenia	22 (35.5)
Osteoporosis	11 (17.7)
**FRAX Hip fractures**	
Low risk	53 (85.5)
High risk	9 (14.5)
**FRAX Major fractures**	
Low risk	51 (82.3)
High risk	11 (17.7)

^1^*n* = 55; *%BF* Percentage of total body fat, *ASMI* Appendicular skeletal muscle mass index, *BMD* Bone mineral density, *BMI* Body Mass Index, *CC* Calf circumference, *FRAX* Fracture Risk Assessment Tool, *HGS* Hand grip strength, *TUGT* Timed Up and Go test, *VAT* Visceral adipose tissue, *WC* Waist circumference

In the correlation analysis considering the sarcopenia phenotypes, muscle strength (HGS) showed moderate and inverse correlations with age (*r* = -0.330; *p* = 0.009), FRAX Hip fractures (*r* = -0.384; *p* = 0.002), and FRAX Major fractures (*r* = -0.383; p = 0.002) (Table 4). In addition, HGS showed a positive and weak correlation with body mass (*r* = 0.291; *p* = 0.021) and a moderate one with CC (*r* = 0.301; *p* = 0.017). Muscle quantity (ASMI) had strong correlation with body mass (*r* = 0.704; *p* < 0.001) and BMI (*r* = 0.748; *p* < 0.001), in addition to a moderate one with CC (*r* = 0.613; p < 0.001), WC (r = 0.601; *p* < 0.001), %BF (*r* = 0.407; *p* = 0.002), and VAT (*r* = 0.477; *p* < 0.001). There were still moderate correlations between physical performance (TUGT) and age (*r* = 0.415; *p* = 0.001), FRAX Hip fractures (*r* = 0.370; *p* = 0.003), and FRAX Major fractures (*r* = 0.663; *p* = 0.008) (Table 4).

**Table 4:** Correlation between sarcopenia phenotypes with age, nutritional status, body composition, clinical variables, and risk of fractures in women with breast cancer

Variables (*n* = 62)	HGS		ASMI		TUGT	
	**r**	**p**	**r**	**p**	**r**	**p**
Age (years)	-0.330	**0.009**	0.153	0.264	0.415	**0.001**
Body mass (Kg)	0.292	**0.021**	0.704	** < 0.001**	0.019	0.881
BMI (Kg/m^2^)	0.122	0.344	0.748	** < 0.001**	0.089	0.489
CC (cm)	0.301	**0.017**	0.613	** < 0.001**	-0.027	0.834
WC (cm)	-0.037	0.778	0.601	** < 0.001**	0.241	0.059
Menopause time (years)	-0.126	0.377	0.012	0.936	0.188	0.187
Diagnosis time (months)	0.065	0.615	0.043	0.755	-0.099	0.446
FRAX Hip fractures (%)	-0.384	**0.002**	-0.028	0.838	0.370	**0.003**
FRAX Major fractures (%)	-0.383	**0.002**	-0.031	0.820	0.336	**0.008**
%BF (%)	0.077	0.577	0.407	**0.002**	0.016	0.907
VAT (cm^2^)	-0.006	0.967	0.477	** < 0.001**	0.176	0.204

Pearson's Correlation. *%BF* Percentage of total body fat, *ASMI* Appendicular skeletal muscle mass index, *BMI* Body Mass Index, *CC* Calf circumference, *FRAX* Fracture Risk Assessment Tool, *HGS* Handgrip strength, *TUGT* Timed Up and Go test, *VAT* Visceral adipose tissue, *WC* Waist circumference. Values in bold represent *p* < 0.05

Table 5 presents the multivariate linear regression analysis. In the final model, the variables FRAX Hip fractures and FRAX Major fractures remained associated with muscle strength (HGS). For each increase in the percentage of FRAX Hip fractures and FRAX Major fractures there was a reduction of 1.11 kg/f and 0.60 kg/f of HGS, respectively. Muscle quantity (ASMI) remained associated with body mass. For each increase in body mass, there was an increase of 0.06 kg/m^2^ in the ASMI. Finally, no variable remained associated with physical performance (TUGT) after adjustments for possible potentially confounding variables (data not shown in the table).

**Table 5:** Variables associated with sarcopenia phenotypes in women with breast cancer after multivariate linear regression analysis. Vitória – ES, 2021–2022

**Variables**	**Raw**			**Model 1**				**Model 2**	
**(*n* = 62)**	**Β**	**CI (95%)**	***p* value**	**Β**	**CI (95%)**	***p* value**	**Β**	**CI (95%)**	***p* value**
**HGS**									
FRAX Hip fractures (%)	-1.18	-1.91 – -0.45	0.002	-1.18	-1.91 – -0.45	0.002	-1.11	-1.91—-0.31	0.007
**HGS**									
FRAX Major fractures (%)	-0.63	-1.02—-0.24	0.002	-0.63	-1.02—-0.24	0.002	-0.60	-1.03—-0.17	0.007
**ASMI**									
Body mass (kg)	0.05	0.04 – 0.07	< 0.001	0.06	0.04 – 0.07	< 0.001	0.06	0.04 – 0.07	< 0.001

^1^*n* = 55; *ASMI* Appendicular skeletal muscle mass index, *FRAX* Fracture Risk Assessment Tool, *HGS* Handgrip strength. **Model 1**: adjusted for age, race/color, and physical activity level. **Model 2**: adjusted for age, race/color, physical activity level, time of menopause (years), time of diagnosis (months), clinical stage, and type of treatment

## Discussion

This study found an association between muscle strength and fracture risk in women with breast cancer. Most of the women investigated showed preserved sarcopenia phenotypes and a low risk of fractures. In contrast, most of the group was characterized by excess body fat.

The relationship between sarcopenia phenotypes and bone health is described in the literature. It does, however, remain unexplored in women with breast cancer. Most previous studies have determined fracture risk through fracture incidence in the population evaluated, and there are few studies using FRAX as an assessment tool [19, 20, 38].

We observed that patients with higher fracture risk had lower HGS. Previous studies demonstrate that low HGS is an independent risk factor for fragility fractures and that fractured patients have relatively low HGS [39, 40].

Amarowicz et al. [40] evaluated postmenopausal women with vertebral fractures and observed that patients in the reduced HGS group had more fractures. Thus, muscle strength was considered a potential parameter for use in clinical practice to identify patients at risk for vertebral fractures [40]. Denk et al. [41] performed a systematic review on the association between reduced HGS and hip fracture in the elderly and observed that low HGS was associated with an increased risk of fracture in all studies analyzed.

This indicator is related not only to the risk and incidence of fractures, but also to the recovery of fractured individuals [39]. Patients with higher HGS show better recovery after hip fracture, which suggests that it can be used as a screening tool to identify those who need intensive rehabilitation [42–44]. Such a finding may contribute to the prognosis of these patients, since hip fractures are most serious and cause permanent disability in approximately 20% of survivors, with only about 40% of the afflicted individuals eventually attaining pre-injury function levels [45].

The relationship between reduced HGS and increased fracture risk can be explained by the association between low muscle strength and decreased BMD [46]. The mechanical stress of muscle contraction can contribute to the maintenance of bone mass, as it exerts trophic and adaptive effects. Since HGS is a representative measure of muscle strength in the body, reduced values may indicate not only an increased risk of falling but also a reduction in local muscle strength, which is associated with the occurrence of fractures [46]. In this context, positive associations between back muscle strength and lumbar spine BMD have been reported, as well as an increased risk of vertebral fractures in situations of reduced strength [47].

The association between risk of fractures and muscle strength observed in this study may contribute to the prevention of sarcopenia and fractures in women with breast cancer, especially of the Luminal subtypes, since muscle strength, when reduced, indicates the stage of probable sarcopenia, and HGS and FRAX assess preconditions for these ailments [2, 32]. Sarcopenia is associated with systemic inflammation and with all cancer treatment modalities and may develop equally among breast cancer subtypes [12, 48]. However, patients with hormone receptor-positive tumors (Luminal), which account for approximately 75% of cases, may have a significantly increased fracture risk as they are eligible for endocrine therapy [5, 49]. Estradiol deficiency leads to an imbalance of bone remodeling, which causes bone loss, microarchitectural deterioration, and increased bone fragility. It is also noteworthy that antiestrogenic effects can lead young patients to menopause, which is the main cause of bone loss in women [5].

In the present study, the muscle quantity, measured by the ASMI, was the only sarcopenia phenotype whose fracture risk was not associated in any of the analyses performed. There are controversies regarding the relationship between muscle mass and the risk of falls and fractures [38, 50, 51]. Harvey et al. [38] observed that ASM, obtained by DXA, contributed minimal predictive information for falls and fractures in postmenopausal women. A cohort study in elderly males demonstrated that ASM and tests of muscle strength and physical performance predicted fracture risk, regardless of FRAX and fall history; however, the inclusion of BMD, either directly or as part of FRAX, attenuated the association between ASM and fracture prediction [50].

Evidence suggests that DXA-derived ASM may not contribute to the prediction of falls and fractures, especially when considering femoral neck BMD, as seen in the present study [50, 51]. This tool does not measure ASM directly, as it reflects a body compartment that is neither fat nor bone, within the upper and lower limbs. In this way, it only estimates muscle mass, which ends up including skin, connective tissues, and water, in addition to minerals, proteins, non-fatty lipids, and soft tissue carbohydrates [50–52]. In addition, soft tissue can influence the measurement of BMD by DXA, since its mass is incorporated into BMD calculation equations [50].

The technical aspects of DXA also help to understand the positive association between body mass and ASMI found in the present study, despite the predominance of excess body fat in the population evaluated. Tissues quantified by DXA (bone mass, lean mass, and fat mass) are components of total body mass and their values are derived from it [51, 52].

After insertion of adjustment variables, TUGT did not remain associated with the risk of fracture obtained by FRAX. According to the EWGSOP definition, TUGT is an indicator of the severity of sarcopenia, which means it should be compromised after reduced strength and muscle mass [2]. Such results were not observed, since the participants showed a predominance of adequate HGS, ASMI, and TUGT, which may have contributed to the loss of association between TUGT and fracture risk. This finding demonstrates that, in this population, age was the most important factor for physical performance, which is expected [53].

The high prevalence of adequate sarcopenia phenotypes and the low risk of fractures in the sample evaluated can be explained by most patients not having started treatment, in addition to them all being free of endocrine therapy. In fact, it is known that breast cancer treatment is one of the main factors that lead to muscle tissue depletion and increased risk of fractures [48]. Nevertheless, a portion of the population analyzed already had a high risk of fractures and probable sarcopenia, in addition to compromised BMD (osteopenia and osteoporosis). Such findings demonstrate the importance of early assessment of these conditions in women with breast cancer, especially the Luminal subtypes, since they can be worsened after treatment [48].

The impairment of sarcopenia phenotypes and the increased risk of fractures are also influenced by sociodemographic characteristics and habits and lifestyle [54, 55]. Age is a recognized risk factor for both conditions, since aging is characterized by a reduction in muscle and bone mass [54, 55]. In the population studied here, age was inversely associated with muscle strength and positively so with the duration of the TUGT, which indicates bad physical performance.

Similarly, excessive alcohol consumption impairs protein synthesis, and exposure of muscle tissue to ethanol results in autophagy, while smoking can increase muscle fatigue, leading to protein metabolism disorders [55]. On the other hand, the practice of physical activity is useful for the recovery of mitochondrial metabolic function, reducing the expression of catabolic genes and increasing protein synthesis [55].

Regarding the influence of lifestyle habits on fracture risk, there seems to be a decrease in bone formation in individuals with excessive alcohol consumption, in addition to a greater risk of falling when drunk [56]. As for smoking, the influence on bone health occurs through different mechanisms. Among these are impaired calcium absorption and vitamin D metabolism, involved in bone formation, and increased levels of free radicals, not to mention nicotine itself, which can reduce estrogen production in women [56]. In this study, we did not consider alcohol consumption and smoking habits in the adjusted models, since they are part of the FRAX assessment items. However, the level of physical activity was included as an adjustment variable, given its importance for the outcome evaluated.

In our study, most women had never used alcohol or cigarettes, which may have contributed to the low prevalence of impaired sarcopenia phenotypes and low risk of fractures. However, most were insufficiently active. The IPAQ, the instrument used to measure this variable, takes into account the performance of activities in the last week, and after diagnosis, patients often reduce daily activities, in addition to often receiving a recommendation to rest during chemotherapy and radiotherapy, and after surgical treatment [34]. These factors may have contributed to the low prevalence of sufficiently active women observed.

As for clinical features, Rathnayake et al. [57] observed significant differences between pre- and post-menopausal women in the phenotypes of sarcopenia – muscle strength, assessed by the HGS test, amount of muscle, measured by the ASMI, and physical performance, obtained by gait speed – and in BMD, in which post-menopausal women had worse results. However, there was no correlation between sarcopenia phenotypes and time of menopause in the population evaluated. We hypothesize that this result may be associated with the predominance of overweight and adequacy of the sarcopenia phenotypes found.

Clinical factors related to breast cancer also influence the risk of fractures, since the prescribed treatment depends on the histological type and molecular subtype of breast cancer (Luminal A, Luminal B, HER2 + , and triple-negative) [58]. Patients in the middle (IIB) or advanced (IIIA, IIIB) clinical stage are the most affected in relation to BMD, probably because of the increase in the production of pro-inflammatory cytokines by the tumor and as a response of the organism, which increases bone resorption [58]. Such factors still exert catabolic effects on muscle tissue, which can compromise muscle strength and muscle mass [3, 4].

Much like sociodemographic and lifestyle variables, time of menopause and clinical variables, such as time of diagnosis, clinical staging, and type of treatment, did not interfere in the association between muscle strength and fracture risk in the women evaluated.

As for nutritional status variables, it has been reported that anthropometric measurements can be positively associated with sarcopenia phenotypes, since they are not able to compartmentalize the constituents of total body mass [57]. On the other hand, body composition parameters are closely related to this disease since it involves the reduction of skeletal muscle mass. Fat mass, when elevated, can promote fat infiltration into skeletal muscle, negatively affecting muscle quality [2].

Muscle mass also exerts a protective effect against increased fracture risk, through molecular signaling and regulation of hormone levels and bone anabolic factors [59]. While the accumulation of fat promotes the differentiation of adipocytes, it induces the secretion of hormones (adiponectin, leptin, and sex hormones) and produces pro-inflammatory cytokines, which modulate the osteoblast-osteoclast interaction, given that excess visceral fat induces greater inflammation, associated with osteoclastogenesis and bone loss [60].

This study observed a positive correlation between the variables of the fat mass compartment and ASMI, which can be explained by the limitation of DXA in measuring muscle mass, as discussed above, as well as in quantifying VAT separately. DXA assesses VAT by estimating the amount of subcutaneous fat in the android region, which is subtracted from the total fat mass of this region, thus limiting its interpretation [52]. Another point to be discussed is the inability of DXA to differentiate the amount of fat infiltrated in the muscle from the total muscle mass [61].

As a contribution, this study demonstrated a relationship between HGS and FRAX, which optimizes the patient assessment process and supports early interventions and strategies, based on simple tools suitable for inclusion in assessment and clinical follow-up protocols. Compared to other tools that evaluate sarcopenia phenotypes, HGS is easy to use in clinical practice, in addition to being low-cost, portable, and not requiring specialized equipment and professionals. Because of these characteristics, its use as part of the routine is recommended in cancer patients [62].

Thus, our results suggest that serial HGS measurements may help to identify women with breast cancer at high risk of fractures. Longitudinal studies are needed to determine whether incorporating muscle strength measures into fracture risk assessment tools would improve predictive accuracy.

This study has some limitations. It has a small sample, not representative of the general population of women with breast cancer, as it was carried out with a group of women from a single outpatient clinic, from the secondary service of the public health system. It does, however, have a homogeneous and controlled sample, which contributes to the validity of the results presented here. In addition, the proposal to assess the relationship between sarcopenia phenotypes and fracture risk in women with breast cancer is an innovative one, not yet described in this population. Another limitation is the possibility of overestimating sarcopenia phenotypes, since there are no cutoff points for these parameters in the literature for cancer patients.

## Conclusions

Low muscle strength was the sarcopenia phenotype that remained associated with fracture risk in women with breast cancer, independently of sociodemographic factors, level of physical activity, and clinical factors. The inclusion of this parameter in the care and follow-up protocols of women with breast cancer may point to the risk of fractures, in addition to probable sarcopenia.

## Data Availability

The datasets used and analyzed during the current study are available from the corresponding author upon reasonable request.

## Abbreviations

CTIBL: Cancer Treatment-Induced Bone Loss
AI: Aromatase Inhibitors
BMD: Bone Mineral Density
FRAX: Fracture Risk Assessment Tool
EWGSOP: European Working Group on Sarcopenia in Older People Revised
HGS: Handgrip strength
ASMI: Appendicular skeletal muscle mass index
TUGT: Timed Up and Go test
ASHT: American Society of Hand Therapists
ASM: Appendicular skeletal muscle mass
DXA: Dual energy x-ray absorptiometry
NHANES: National Health and Nutrition Examination Survey
ISCD: International Society for Clinical Densitometry
SBDens: Brazilian Society for Clinical Densitometry
CC: Calf circumference
WC: Waist circumference
BMI: Body mass index
WHO: World Health Organization
PAHO: Pan American Health Organization
%BF: Percentage of total body fat
VAT: Visceral adipose tissue
ABRASSO: Brazilian Association of Bone Assessment and Osteometabolism
IPAQ: International Physical Activity Questionnaire
SPSS: Social Package Statistical Science
